# Supplementary material for: RAD54 promotes alternative lengthening of telomeres by mediating branch migration
Source: EMBO Rep. 2020 Apr 26;21(6):e49495. doi: 10.15252/embr.201949495 (PMC7271314; doi:10.15252/embr.201949495)
Supplement: Supplementary file 1 — Expanded View Figures PDF [file EMBR-21-e49495-s001.pdf]

Expanded View Figures

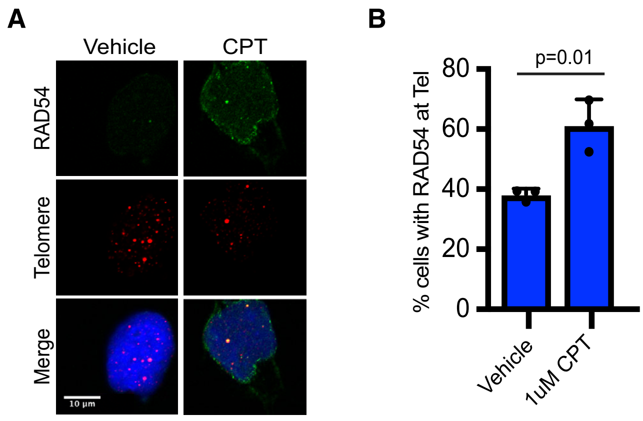

Figure EV1. Genome-wide DNA damage enriches RAD54 at telomeres.

A Combined IF and DNA FISH for RAD54 and telomeres in SaOS2 treated with vehicle (DMSO) or camptothecin (CPT) 1 µM for 1 h immediately prior to staining. Scale bars = 10 µm.

B Quantification of A. A cell was counted as positive if it contained at least 1 colocalization event between RAD54 and telomere. At least 100 cells were counted per repeat, $n = 3$. Values shown are mean ± SD. Values were compared using unpaired two-tailed Student's *t*-test.

© 2020 The Authors

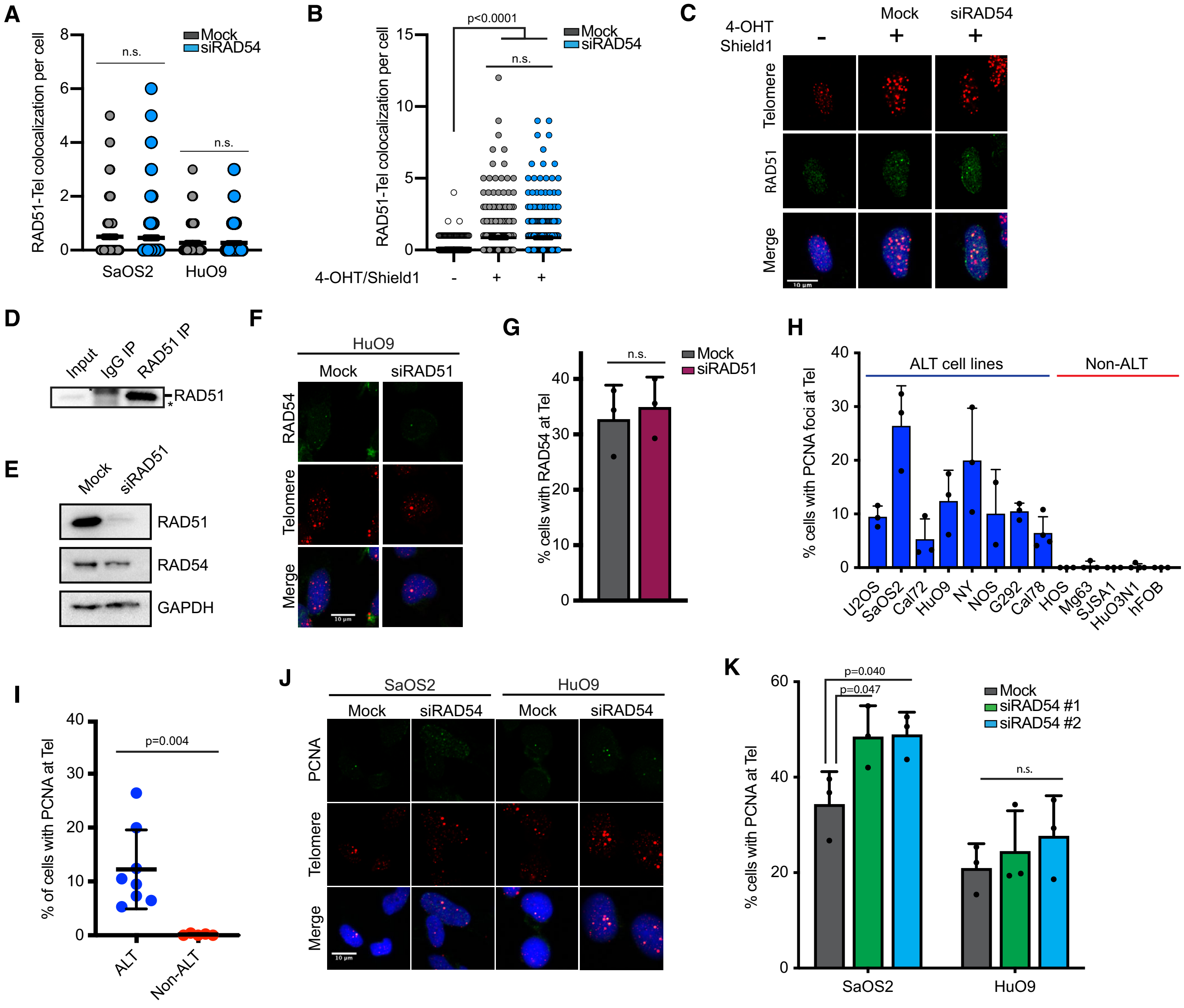

Figure EV2. RAD54 is dispensable for early synaptic events at ALT telomeres.

A Quantification of data shown in Fig 2A. Values shown are mean ± SEM, $n = 3$ with at least 100 cells quantified per repeat. Values were compared using two-way ANOVA followed by Sidak's test.

B Quantification of RAD51 localization with telomeres in U2OS-TRF1-FOK1 cells. To induce TRF1-FOK1 cells were treated with 40 ng/ml doxycycline for 16 h and then 1 μM Shield-1 and 1 μM 4-OHT for 4 h. Values shown are mean ± SEM, $n = 3$ with at least 100 cells quantified per repeat. Values were compared using Kruskal–Wallis test followed by Dunn's test.

C Representative images of combined IF and DNA FISH for RAD51 (IF) and telomeres (FISH) in U2OS-TRF1-FOK1-WT cells that were either mock-transfected or transfected with RAD54 siRNA. Scale bar = 10 μm.

D Western blot of RAD51 immunoprecipitation from HuO9 cells. Blot was probed for RAD51. Asterisk indicates non-specific band in IgG pull down.

E Western blot confirming RAD51 knockdown from samples used in Fig EV2F and G.

F Representative image from combined IF and DNA FISH for RAD54 at telomeres in HuO9 cells transfected with 20 nM siRAD51 for 48 h. Scale bars = 10 μm.

G Quantification of data from F. Cells were counted positive if they had at least 1 colocalization event between RAD54 and telomeres. At least 100 cells per condition were counted per repeat. Values shown are mean ± SD, $n = 3$. Values were compared using unpaired two-tailed Student's *t*-test.

H Quantification of focal accumulation of PCNA at telomeres across a panel of ALT and non-ALT cell lines. Cells were counted positive if they had at least 1 PCNA foci that colocalized with telomeres. Pan-nuclear staining was excluded. At least 100 cells were counted per cell line per repeat. Values shown are mean ± SD, $n = 3$.

I Data from H graphed according to ALT status. Each dot represents the mean value for one cell line. Values shown are mean ± SD. Data were compared using unpaired two-tailed Student's *t*-test.

J Representative images from combined IF and DNA FISH for PCNA at telomeres in HuO9 or SaOS2 transfected with 20 nM siRAD54#2 for 48 h. Scale bars = 10 μm.

K Quantification of data from J. HuO9 or SaOS2 transfected with 20 nM siRAD54#1 or 20 nM siRAD54#2 for 48 h. Data were quantified as in H. Values shown are mean ± SD, $n = 3$ biological replicates. Values were compared using standard two-way ANOVA followed by Dunnet's multiple comparison test.

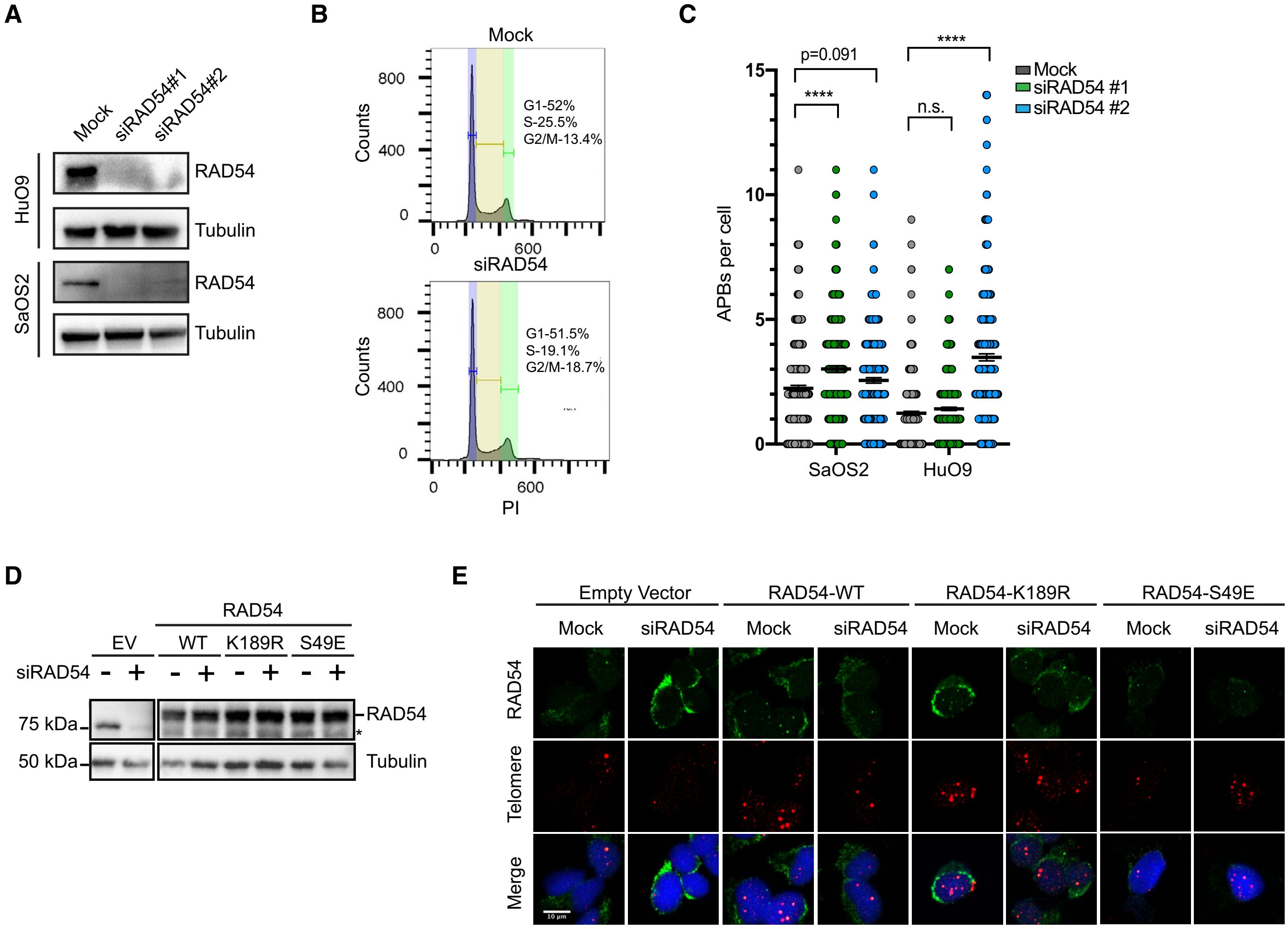

Figure EV3. RAD54 regulates ALT activity without altering cell cycle.

- A Representative Western blot for RAD54 in HuO9 and SaOS2 cells transfected with 20 nM siRAD54#1 or siRAD54 #2 for 48 h.
- B Cell cycle profile of HuO9 cells mock-transfected or transfected with 20 nM siRAD54 #2 for 48 h.
- C Quantification of the number of APB per cell in HuO9 or SaOS2 cells that were either mock-transfected or transfected with 20 nM siRAD54#1 or 20 nM siRAD54#2 for 48 h. At least 100 cells were counted per condition per repeat, $n = 3$. Values shown are mean ± SEM. Data were analyzed using two-way ANOVA followed by Sidak's multiple comparison test. **** indicates $P < 0.0001$.
- D Representative Western blot for RAD54 on samples from Figs 3G and H, and EV3E. Overexpressed RAD54 runs as a doublet. Top band is the expected molecular weight of SFB-RAD54. Asterisk indicates band that is a cleavage product of overexpressed RAD54. Tubulin used as a loading control.
- E Representative images of IF-DNA FISH showing RAD54 at the telomeres using SFB-RAD54 constructs and siRAD54#2. HuO9 cells were forward transfected with 2 μg empty vector (EV), RAD54-WT, RAD54-K189R, or S49E construct. After 24 h, cells were transfected with 20 nM siRAD54#2 for 48 h prior to IF staining. Scale bars = 10 μm.

 © 2020 The Authors

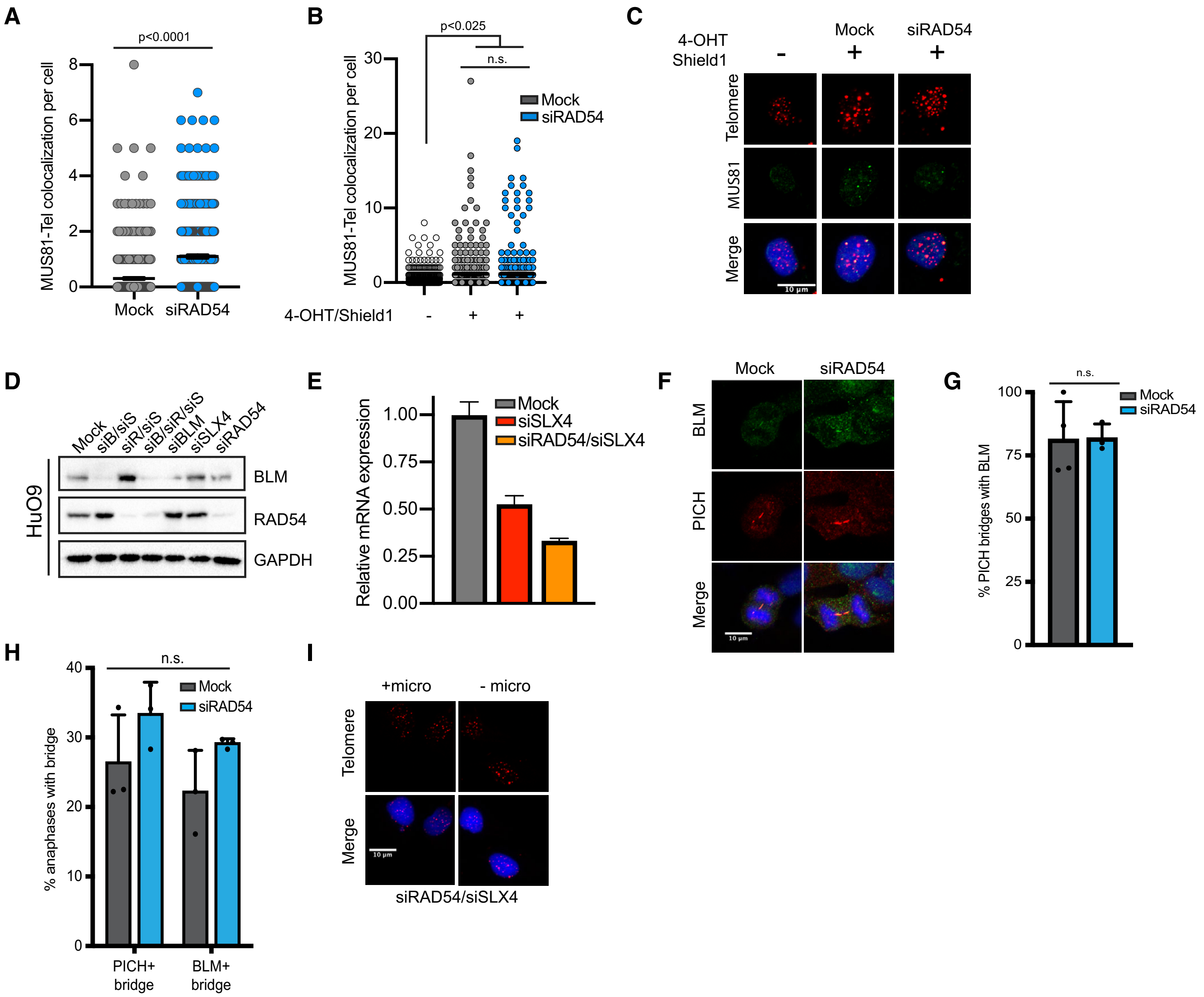

Figure EV4. RAD54 limits UFB formation and genome instability.

A Quantification of data shown in Fig 4C. Values shown are mean ± SEM, $n = 3$ with at least 100 cells quantified per repeat. Values were compared using Mann–Whitney test.

B Quantification of MUS81 localization with telomeres in U2OS-TRF1-FOK1 cells. To induce TRF1-FOK1 cells were treated with 40 ng/ml doxycycline for 16 h and then 1 μM Shield-1 and 1 μM 4-OHT for 4 h. Values shown are mean ± SEM, $n = 3$ with at least 100 cells quantified per repeat. Values were compared using Kruskal–Wallis test followed by Dunn's test.

C Representative images of combined IF and DNA FISH for MUS81 (IF) and telomeres (FISH) in U2OS-TRF1-FOK1-WT cells that were either mock-transfected or transfected with RAD54 siRNA. Scale bar = 10 μm.

D Representative Western blot validating knockdown of BLM and RAD54 in HuO9. Cells were transfected with siRNAs for 72 h prior to Western blot.

E RT–qPCR data for SLX4 knockdown in HuO9 cells. Cells were transfected with siSLX4 or siRAD54#2 combined with siSLX4 for 48 h. Data shown are mean ± SEM for technical triplicates.

F Representative images of ultra-fine bridge (UFB) detection staining for PICH and BLM on HuO9 cells. HuO9 cells were transfected with siRAD54#2 for 72 h. Scale bars = 10 μm.

G Quantification of data shown in F. At least 60 total PICH bridges were counted in each condition over 3 biological replicates. A PICH bridge was considered positive for BLM if there was evidence of BLM staining over all or part of the PICH-coated bridge. Values shown are mean ± SD. Values were compared using two-tailed unpaired Student's *t*-test.

H Quantification of data shown in F. At least 90 total anaphases were scored over 3 biological replicates. An anaphase was considered positive if it contained 1 or more PICH or BLM bridges. Values shown are mean ± SEM. Conditions were compared using a two-way ANOVA followed by Tukey's multiple comparison test.

I Representative DNA FISH images of HuO9 cells transfected with siRAD54#2 and siSLX4 for 48 h with and without a micronucleus containing telomere FISH signal (indicated by white arrow). From data quantified in Fig 4K. Scale bars = 10 μm.
